# Rapid Screening and Structural Characterization of Antioxidants from the Extract of *Selaginella doederleinii* Hieron with DPPH-UPLC-Q-TOF/MS Method

**DOI:** 10.1155/2015/849769

**Published:** 2015-02-22

**Authors:** Gang Wang, Shun Yao, Xiu-Xiu Zhang, Hang Song

**Affiliations:** ^1^Zunyi Medical College, Zunyi, Guizhou 563003, China; ^2^Sichuan University, Chengdu, Sichuan 610000, China

## Abstract

2,2-Diphenyl-1-picrylhydrazyl-ultra-high performance liquid chromatography-Q-time-of-flight mass spectrometry (DPPH-UPLC-Q-TOF/MS), as a rapid and efficient means, now was used for the first time to screen antioxidants from *Selaginella doederleinii*. The nine biflavone compounds were screened as potential antioxidants. The biflavones were structurally identified and divided into the three types, that is, amentoflavone-type, robustaflavone-type, and hinokiflavone-type biflavonoids. Among the compounds bilobetin (3) and putraflavone (8) were found from *Selaginella doederleinii* for the first time and others including amentoflavone (1), robustaflavone (2), 4′-methoxy robustaflavone (4), podocarpusflavone A (5), hinokiflavone (6), ginkgetin (7), and heveaflavone (9) were identified previously in the plant. Moreover, nine biflavones possessed a good antioxidant activity via their DPPH free radical scavenging. It demonstrates that DPPH-UPLC-Q-TOF/MS exhibits strong capacity in separation and identification for small molecule. The method is suitable for rapid screening of antioxidants without the need for complicated systems and additional instruments.

## 1. Introduction

*Selaginella doederleinii* Hieron, as a traditional Chinese medicine, is a well-known perennial pteridophyte plant growing in South and Southwestern China at the low altitude. It has the function of eliminating wind, scattering frigidity, detumescence, and treating cough [1]. In the reported research, different types of compounds from* Selaginella doederleinii*, including biflavonoid, alkaloid, xylogen, sterol, and organic acid, have been exhibited [2–6].

**Figure 1:**
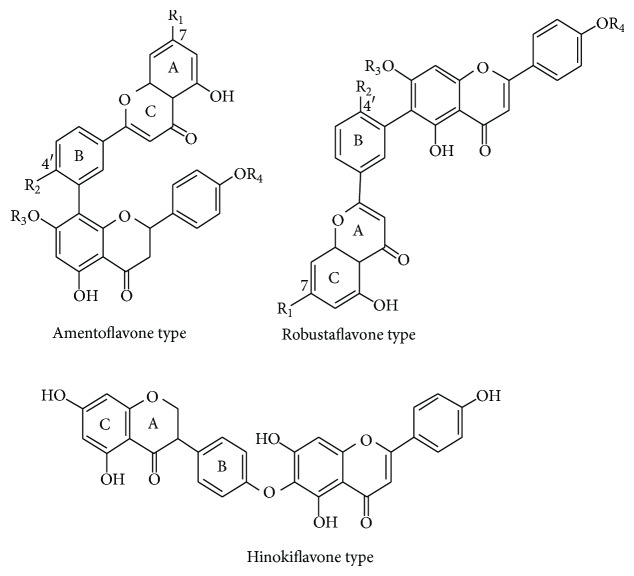
Chemical structures of bioflavonoids in* Selaginella doederleinii*.

Some researches [7] have proposed that the potential antioxidants in* Selaginella doederleinii* are biflavonoids, including amentoflavone, robustaflavone, heveaflavone, and robustaflavone 4′,  4′′′-O-dimethyl ether (see Figure 1). Most biflavonoids, just like apigenin and its methoxyl/hydroxyl substituents, have prominent antioxidant characteristics and show a wide range of biological activity, such as cancer chemopreventive properties, anti-inflammatory, antimicroorganism, and antioxidative effects [8–13].

Traditional screening for antioxidant activity components from natural plants is a time-consuming, inefficient, and costly method, which cannot meet the need of the development of modern scientific research [14]. Therefore, it is necessary to find out more new and efficient online techniques to search the active compounds from natural products.

This study aimed to develop a rapid, efficient, and stable method to screen and identify the main antioxidative components in the extracts from* Selaginella doederleinii*. The online 2,2-diphenyl-1-picrylhydrazyl-ultra-high performance liquid chromatography-Q-time-of-flight mass spectrometry (DPPH-UPLC-Q-TOF/MS) technique was used for the first time to screen and identify antioxidants from ethyl acetate extract in this study. Nine active biflavonoids were yielded and included amentoflavone (1), robustaflavone (2), bilobetin (3), 4′-methoxy robustaflavone (4), podocarpusflavone A (5), hinokiflavone (6), ginkgetin (7), putraflavone (8), and heveaflavone (9). Among them, compounds 3 and 8 in this study were first reported from* Selaginella doederleinii*.

## 2. Experimental

### 2.1. Chemicals and Materials

Herbal materials were identified as* Selaginella doederleinii* by Vice Professor Yang Jianwen from the Department of Pharmacy in Zunyi Medical College. A voucher specimen (20120923) has been preserved in the Key Laboratory of Natural Pharmaceutical Chemistry, Zunyi Medical College.

2,2-Diphenyl-1-picrylhydrazyl (DPPH) was purchased from Sigma Pure Chemical Industry (Berlin, Germany). Butylated hydroxytoluene (BHT), ascorbic acid, and quercetin were purchased from BDH Company (Poole, England). Acetonitrile of HPLC grade was provided by Merck Chemical Industry (Darmstadt, Germany). All other chemicals and solvents used were of analytical grade.

### 2.2. Preparation of* Selaginella doederleinii* Hieron Extract

100.0 g of dried* Selaginella doederleinii* was powdered and extracted three times (each for 2 h) with 95% ethanol (1.0 L). The extracting solution was filtrated, merged, and centrifuged at 4000 rpm under 5°C. The supernatant was concentrated and dried under vacuum. Then 18.37 g extracts were finally obtained from* Selaginella doederleinii*, which were then dissolved in warm water and used for liquid-liquid extraction with petroleum ether, ethyl acetate, and n-butyl alcohol successively. The three fractions were concentrated, dried, and obtained 0.52 g, 4.19 g, and 6.64 g, respectively. All fractions were stored in refrigerator (4°C) for further use.

### 2.3. Evaluation of Antioxidant Activity

DPPH is a very stable nitrogen center of free radicals. The DPPH oxidative assay is widely used for quantitative determination of antioxidant capacity in biological samples and food [15]. In short, each of polarity fractions was dissolved in methanol (4 mg·mL^−1^ in methanol) and diluted different concentrations sample (0.08~0.14 mg·mL^−1^ in methanol). 1.0 mL of the sample was mingled with 4.0 mL DPPH solution (0.6 mg·mL^−1^ in methanol). The mixture was completely shaken, reacted, and left at room temperature in the dark for 60 min. The absorbance was measured at 517 nm in an ultraviolet spectrophotometer (Shimadzu UV-Vis 160A, Japan). The percentage of DPPH inhibition was calculated by using the following equation:

(1) $$\begin{array}{c} I\left(\%\right)=\left[\frac{\left(A_{b}-A_{s}\right)}{A_{b}}\right]\times 100\%, \end{array}$$

where *I* is the inhibition percentage, *A*_*b*_ is the absorbance of the blank sample, and *A*_*s*_ is the absorbance of the test sample. Sample concentration providing 50% inhibition (IC_50_) was calculated by plotting the inhibition percentage against different concentrations sample. BHT, ascorbic acid, and quercetin were used as positive controls. All samples of the test were run in triplicate and the average value was calculated.

### 2.4. DPPH–UPLC-Q-TOF-MS Experiment

The extract from* Selaginella doederleinii* was mingled with DPPH solution of different concentrations (0.16, 0.32, and 0.48 mM·L^−1^) at the ratio of 1 : 1 (w/v). The mixture was completely shaken, produced reaction, and was left at room temperature in the dark for 60 min. Then, the mixture was filtered through a 0.45 *μ*m pinhole-membrane filter and injected into the UPLC system. For the control sample methanol was added instead of DPPH to the extract from* Selaginella doederleinii*.

The mixture and control samples were analyzed in an Acquity UPLC system (Waters Corp., Milford, MA, USA) equipped with ultraviolet (UV) and a BEH C18 (100 × 2.1 mm, 1.7 *μ*m). The detection wavelength was 330 nm. Formic acid (0.1%) and acetonitrile were, respectively, used as the mobile phases A and B. The solvent gradient was used as follows: 30–80% B between 0 and 25 min, 80–95% B between 25 and 27 min, and 95–100% B between 27 and 30 min. The flow rate was set at 0.6 mL/min at 30°C and the injection volume of the sample was 5 *μ*L. In comparison to the UPLC chromatographic profiles of the reaction samples and the control samples, the main antioxidants could be screened from* Selaginella doederleinii*.

The molecular weight and structure of the screened antioxidants were identified by Q-TOF/MS. High-purity nitrogen was utilized as a nebulizer, auxiliary gas was set at a 2500 L/h, and flow rate was set at 350 L/h. Argon was used as the collision gas at a flow rate of 50 L/h under 1000 degrees. Electrospray ionization (ESI) capillary voltage was set at +3.0 kV for positive ion mode. The sample cone voltage was set at 33 V and the collision energy (CE) was set at 2.5 eV. The mass scan was in the range of 200–1500 *m*/*z* and sweeping time was 1 s.

## 3. Results and Discussion

### 3.1. Antioxidant Activity of Different Fractions

Ethanol extract from* Selaginella doederleinii* was partitioned with different polarity solvents successively. Antioxidant activity of petroleum ether, ethyl acetate, and n-BuOH fractions was then evaluated by DPPH radical scavenging activity. As shown in Table 1, compared with BHT, ascorbic acid, and quercetin, ethyl acetate fraction has the very good antioxidant activity among different polarity fractions. Therefore, the antioxidants were screened and identified from ethyl acetate fraction of* Selaginella doederleinii* with the online DPPH-UPLC-Q-TOF/MS assay.

### 3.2. DPPH–UPLC Analysis for Screening of Main Antioxidants

**Figure 2:**
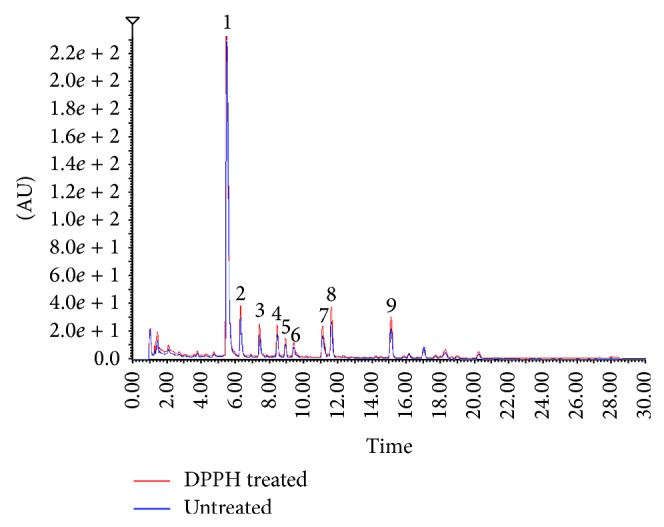
Chromatograms of the ethyl acetate fraction of* Selaginella doederleinii* before and after reaction with DPPH radicals.

According to the UPLC conditions in Section 2.4, the quick analysis results of the potential antioxidants from* Selaginella doederleinii* are shown in Figure 2. An antioxidant and a DPPH radical could mingle and produce the oxidation reaction. After that, the oxidation would change the molecular structure of the antioxidant [16]. It was concluded that the peak areas (PAs) of the antioxidant would obviously decrease in the UPLC chromatogram after reaction with DPPH. But the PAs of the compounds without antioxidant effect witnessed almost no change. Therefore, the untreated and DPPH-treated ethyl acetate fraction from* Selaginella doederleinii* were analyzed by the optimized UPLC separation conditions as stated. Through the comparison of the UPLC chromatograms of untreated and DPPH-treated samples, the PAs of nine main peaks were all decreased significantly. And nine peaks were detected and isolated from the sample. Their retention time was 5.25, 6.19, 7.28, 8.32, 8.81, 9.27, 10.92, 11.47, and 14.92 min, respectively (see Figure 2). The results indicated that these nine peaks were all antioxidants existing in the ethyl acetate fraction.

In order to estimate the radical scavenging capacity of the nine antioxidants, the ethyl acetate fraction was reacted with DPPH of different concentrations. PAs of nine peaks of untreated sample were set as 100% and the relative PAs of the sample that were reacted with DPPH of different concentrations were calculated. As the result, the PAs of nine peaks all decreased with the increase of DPPH concentration. At first peak 2 decreased more sharply than the other eight peaks. But with the increase of DPPH concentration, peak 6 decreased the most among the nine peaks (see Table 2). The results indicate that peak 6 has the strongest radical scavenging capacity and peaks 5, 3, 7, 8, 9, 1, 4, and 2 follow suit.

### 3.3. Identification of Main Natural Antioxidants

The nine compounds had been identified as biflavonoids. The pure biflavonoid compounds were divided into the three types, which were the amentoflavone-type, robustaflavone-type, and hinokiflavone-type biflavonoids, respectively. The ionization of these compounds was included in both the positive and negative ESI ion modes. The positive ion mode provided more rich, accurate, and specific signals than the negative ion mode. So, positive ion mode was mainly adopted to analyze the nine compounds. The characteristic fragment ion peaks observed by the cleavage of [M+H]^+^ ions of the three types of biflavonoids were shown in Table 3.

#### 3.3.1. Fragmentation Behavior of Amentoflavone-Type Biflavonoids

Compound 1 showed the quasimolecular ion [M+H]^+^ at *m*/*z* 539.0956. It is two significant fragmentations cracking way. The most useful fragmentation about compound 1 produces RDA cleavage at position 1/3 of the C-ring on flavonoid part II. The fragmentation pathway leads to the [M+H-C_8_H_6_O]^+^ at *m*/*z* 421. At the same time, after [M+H]^+^ can also lose a H_2_O molecule and undergo RDA cleavage at the same 1/3 position, the fragmentation pathway leads to the [M+H-H_2_O-C_8_H_6_O]^+^ at *m*/*z* 403. And then, it further loses a series of CO molecules continuously and gives rise to the fragment ions at *m*/*z* 375 and 347. After undergoing the cleavage at position 0/4 of the C-ring on flavonoid part II, another important pathway can lead to the [M+H-C_9_H_6_O_3_]^+^ at *m*/*z* 377, [M+H-C_9_H_6_O_3_-C_2_H_2_O]^+^ at *m*/*z* 335, and [M+H-C_9_H_6_O_3_-C_2_H_2_O-CO]^+^ at *m*/*z* 307, continuously.

So compound 1 was determined as amentoflavone on the basis of previous deduction and literature [17]. Moreover, the [C_7_H_6_O_2_]^+^ at *m*/*z* 121 and the [C_7_H_4_O_4_]^+^ at *m*/*z* 153 are important ions, because they provide information whether there is a substituent at B-ring C4 on flavonoid part II and at A-ring C7 from flavonoid part I.

**Figure 3:**
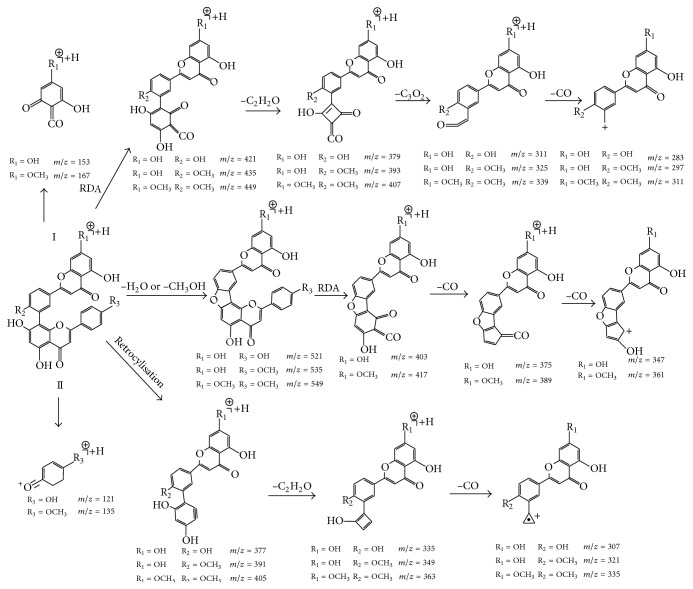
Proposed fragmentation pathways of amentoflavone-type biflavonoid on the basis of their MS^2^ and MS^3^ spectra.

Compounds 3, 5, 7, 8, and 9 exhibited the quasimolecular ion [M+H]^+^ at *m*/*z* 553.1110, 553.1086, 567.1273, 567.1283, and 581.1434, respectively. Due to involving the cleavage of the C-ring at positions 1/3 and 0/4 on flavonoid part II, observations of the five compounds fragmentations cleavage pathway indicate that compounds 3, 5, 7, 8, and 9 clearly belong to an amentoflavone-type biflavonoid (see Figure 3 and Table 3).

A series of prominent ions at *m*/*z* 435, 403 (base peak), 347, 377, 335, and 307 were also found in compound 3 ion mass spectra of [M+H]^+^. What is more, other compound 3 characteristic ions were also found such as [M-C_8_H_6_-C_2_H_2_O-C_3_O_2_-CO]^+^ at *m*/*z* 283, [C_7_H_4_O_4_]^+^at *m*/*z* 153, and [C_8_H_7_O_2_]^+^ at *m*/*z* 135. The analysis results indicate that no substituent is observed on flavonoid part II. But a methoxy group at position C4 of the B-ring on flavonoid part I is observed. So compound 3 was considered a bilobetin.

A series of prominent ions at *m*/*z* 421, 403 (base peak), 347, 377, 335, and 307 were observed in compound 5 ion mass spectra of [M+H]^+^. In addition, compound 5 ions were also found such as [M-C_8_H_6_-C_2_H_2_O-C_3_O_2_-CO]^+^ at *m*/*z* 283, [C_7_H_4_O_4_]^+^ at *m*/*z* 153, and [C_8_H_7_O_2_]^+^ at *m*/*z* 135. According to analysis results above, no substituent is found on flavonoid part I and a methoxy group is observed at position C4 of the B-ring on flavonoid part II. So compound 3 was identified as a podocarpusflavone A.

A series of prominent ions at *m*/*z* 549, 417 (base peak), 391, 361, and 311 were observed in compound 7 ion mass spectra of [M+H]^+^. Moreover, other compound 7 ions were also observed such as [C_8_H_6_O_4_]^+^ at *m*/*z* 167 and [C_7_H_6_O_2_]^+^ at *m*/*z* 121. The results show that no substituent is found on flavonoid part II. But a methoxy group at position C4 of the B-ring on flavonoid part I and a methoxy group at position C7 of A-ring on flavonoid part I are found. So compound 7 was identified as ginkgetin.

A series of prominent ions at *m*/*z* 435, 417 (base peak), 389, 361, and 321 were observed in compound 8 ion mass spectra of [M+H]^+^. Furthermore, other compound 8 ions were also observed such as [M-C_8_H_6_-C_2_H_2_O-C_3_O_2_-CO]^+^ *m*/*z* 297, [C_8_H_6_O_4_]^+^ at *m*/*z* 167, and [C_8_H_7_O_2_]^+^ at *m*/*z* 135. According to analysis results above, it is indicated that this molecule has a methoxy group at position C4 of the B-ring on flavonoid part II and a methoxy group at position C7 of A-ring on flavonoid part I. So compound 8 was identified as putraflavone.

A series of prominent ions at *m*/*z* 549, 417 (base peak), 389, 361, and 335 were observed in compound 9 ion mass spectra of [M+H]^+^. What is more, other compound 9 ions were also observed such as [M-C_8_H_6_-C_2_H_2_O-C_3_O_2_-CO]^+^ at *m*/*z* 311, [C_8_H_6_O_4_]^+^ at *m*/*z* 167, and [C_8_H_7_O_2_]^+^ at *m*/*z* 135. The analysis results above indicate that a methoxy group is observed at position C4 of B-ring on flavonoid part II, a methoxy group is observed at position C7 of the A-ring, and a methoxy group is found at position C4 of the B-ring on flavonoid part I. So compound 9 was considered heveaflavone.

#### 3.3.2. Fragmentation Behavior of Robustaflavone-Type Biflavonoids

Proposed fragmentation pathways of robustaflavone-type biflavonoid were shown in Figure 3. Compound 2 exhibited the quasimolecular ion [M+H]^+^ at *m*/*z* 539.0977. Compared with that of amentoflavone-type biflavonoids, they are two important and different fragmentations cleavage pathway. One important fragmentation involves RDA cleavage at position 1/3 of the C-ring on flavonoid part I. The fragmentation pathway gives rise to the [M+H-C_7_H_4_O_4_]^+^ at *m*/*z* 387. Getting involved with the cleavage at position 1/4 of the C-ring on flavonoid part I, the other significant pathway arouses the [M+H-C_6_H_4_O_3_]^ +^ at *m*/*z* 413. What is more, we noted one special electronion at *m*/*z* 270. It displays a cleavage as a connection of the two flavonoid parts. So compound 2 was determined as robustaflavone on the ground of previous deduction and literature [18].

**Figure 4:**
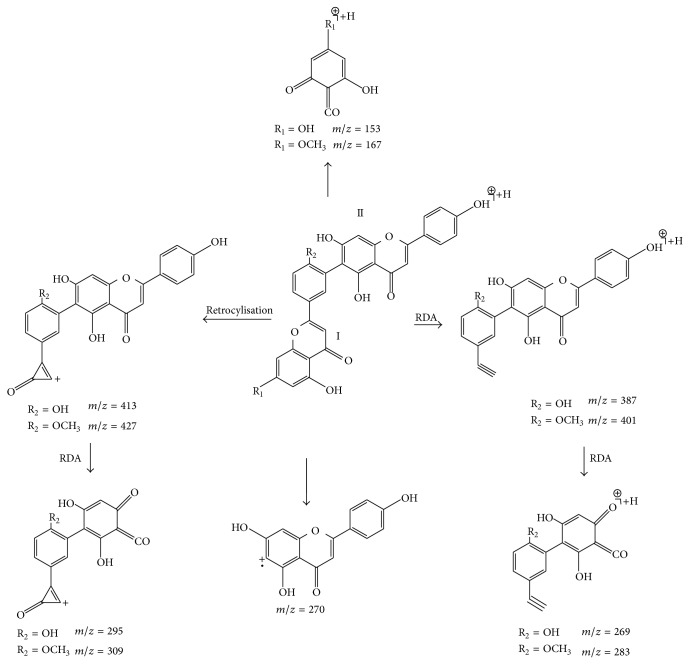
Proposed fragmentation pathways of robustaflavone-type biflavonoid on the basis of their MS^2^ and MS^3^ spectra.

Compound 4 exhibited the quasimolecular ion [M+H]^+^ at *m*/*z* 553.1127. A series of main ions at *m*/*z* 427, 401, 309, and 283 were found in compound 4 ion mass spectra of [M+H]^+^. According to the [M-C_6_H_4_O_4_]^+^ at *m*/*z* 401 and [M-C_5_H_4_O_3_]^+^ at *m*/*z* 427 of compound 4 (see Figure 4), compound 4 is a robustaflavone-type biflavonoid. Because the typical fragmentation pathway of robustaflavone gets the cleavage at positions 1/4 and 1/3 of C-ring on flavonoid part I, other compound 4 characteristic ions are also found such as [C_7_H_4_O_4_]^+^ at *m*/*z* 153. The analysis results indicate that a methoxy group at position C4 of the B-ring on flavonoid part I is observed. So compound 4 was taken as 4′-methoxy robustaflavone.

#### 3.3.3. Fragmentation Behavior of Hinokiflavone-Type Biflavonoids

**Figure 5:**
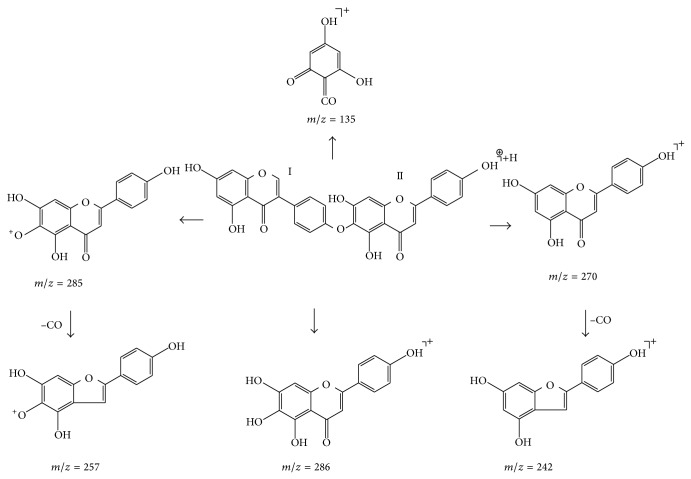
Proposed fragmentation pathways of hinokiflavone.

Proposed fragmentation pathways of hinokiflavone were shown in Figure 5. Compound 6 exhibited the quasimolecular ion [M+H]^+^ at *m*/*z* 539.0963. The most main diagnostic ions are a sequence of ions at *m*/*z* 286, 270, 254, and 242, which root in the rupture of the C-O connection on flavonoid parts I and II (see Figure 5). Furthermore, the predominant ion at *m*/*z* 257 is found by the quasimolecular ion losing flavonoid part I and CO, continuously. So compound 6 was determined as hinokiflavone on the basis of previous deduction and literature [19]. Through the above analysis, the major fragmentation of the hinokiflavone-type biflavonoids shows great difference with the first two types of biflavonoids, because two flavonoid parts of the hinokiflavone type are not a C-C bond but a C-O bond.

## 4. Conclusions

UPLC-DPPH radical spiking test combined with Q-TOF/MS, as a rapid, simple, and economical analysis method with which screening antioxidants from* Selaginella doederleinii* successfully was carried out. The nine biflavone compounds were screened as potential antioxidants. The biflavonoids were structurally identified and divided into the three types, that is, amentoflavone, robustaflavone, and hinokiflavone. Among them, bilobetin and putraflavone were found from* Selaginella doederleinii* for the first time. The identified biflavonoids may contribute via their DPPH free radical scavenging potential to the beneficial antioxidation effects of* Selaginella doederleinii*. It demonstrates that UPLC-Q-TOF/MS assay has a great possibility to become high-advanced method for antioxidants screening and identification of constituents in* Selaginella doederleinii*, and it will be useful to the high-value application of the rich medicinal herbs in China.

## Figures and Tables

**Table 1:** Antioxidant activities of different fractions from *Selaginella doederleinii* in DPPH assay.

Samples	DPPH (IC_50_, *μ*g·mL^−1^)^a^
Petroleum ether fraction	53.1 ± 0.8
Ethyl acetate fraction	12.5 ± 1.6
n-BuOH fraction	29.4 ± 1.1
Ascorbic acid^b^	15.6 ± 1.2
Quercetin^b^	4.7 ± 0.5
BHT^b^	46.8 ± 0.3

^a^Each value is mean ± SD (*n* = 3); ^b^is used as control.

**Table 2:** Relative PAs of nine peaks after being reacted with different concentrations of DPPH.

Number	DPPH concentration	Relative PAs								
		1	2	3	4	5	6	7	8	9
1	Unreacted control sample	100	100	100	100	100	100	100	100	100
2	Reacted with DPPH	73.2 ± 0.6	57.3 ± 1.5	67.7 ± 1.8	67.7 ± 1.1	72.4 ± 0.4	65.8 ± 0.9	78.1 ± 0.9	80.8 ± 1.4	84.3 ± 1.8
	(0.16 mM·L^−1^)									
3	Reacted with DPPH	34.5 ± 1.3	53.6 ± 0.7	42.7 ± 1.9	32.5 ± 1.7	32.3 ± 1.3	10.3 ± 0.4	36.5 ± 0.7	32.8 ± 1.0	36.9 ± 1.2
	(0.32 mM·L^−1^)									
4	Reacted with DPPH	14.6 ± 1.1	20.1 ± 1.4	11.4 ± 0.5	14.9 ± 1.6	10.7 ± 0.8	10.2 ± 1.5	12.1 ± 1.1	12.6 ± 0.6	13.2 ± 0.5
	(0.48 mM·L^−1^)									

Note: PAs stand for peak areas with the unit of mAU ∗ S.

**Table 3:** Identification of 9 biflavonoids from *Selaginella doederleinii* by UPLC/Q-TOF MS/MS.

Peak number	tR (min)	Measured (M+H)^+^	Calculated	ESI-MS/MS (*m*/*z*)	Formula	Identification
1	5.25	539.0956	538.0899	539 [M+H]^+^ 421 [M+H-C_8_H_6_O]^+^ 403 [M+H-C_8_H_6_O-H_2_O]^+^ 377 [M+H-C_9_H_6_O_3_]^+^ 153 [C_7_H_4_O_4_]^+^ 121 [C_7_H_6_O_2_]^+^	C_30_H_18_O_10_	Amentoflavone

2	6.19	539.0977	538.0899	539 [M+H]^+^ 413 [M+H-C_6_H_4_O_3_]^+^ 387 [M+H-C_7_H_4_O_4_]^+^ 270 [M+H-C_15_H_9_O_5_]^+^	C_30_H_18_O_10_	Robustaflavone

3	7.28	553.1110	552.1056	553 [M+H]^+^ 435 [M+H-C_8_H_6_O]^+^ 403 [M+H-C_8_H_6_O-CH_3_OH]^+^ 391 [M+H-C_9_H_6_O_3_]^+^ 297 [M+H-C_8_H_6_O-C_2_H_2_O-C_3_O_2_-CO]^+^ 153 [C_7_H_4_O_4_]^+^ 121 [C_7_H_6_O_2_]^+^	C_31_H_20_O_10_	Bilobetin

4	8.32	553.1127	552.1056	553 [M+H]^+^ 427 [M+H-C_6_H_4_O_3_]^+^ 401 [M+H-C_7_H_4_O_4_]^+^ 270 [M+H-C_15_H_9_O_5_]^+^	C_31_H_20_O_10_	4′-Methoxy robustaflavone

5	8.81	553.1086	552.1056	553 [M+H]^+^ 421 [M+H-C_9_H_8_O]^+^ 403 [M+H-C_9_H_8_O-H_2_O]^+^ 377 [M+H-C_10_H_8_O_3_]^+^ 153 [C_7_H_4_O_4_]^+^ 135 [C_8_H_8_O_2_]^+^	C_31_H_20_O_10_	Podocarpusflavone A

6	9.27	539.0963	538.0899	539 [M+H]^+^ 286 [M+H-C_15_H_9_O_4_]^+^ 270 [M+H-C_15_H_9_O_5_]^+^ 257 [M+H-C_15_H_9_O_4_-CO]^+^ 242 [M+H-C_15_H_9_O_5_-CO]^+^	C_30_H_18_O_10_	Hinokiflavone

7	10.92	567.1273	566.1212	567 [M+H]^+^ 449 [M+H-C_8_H_6_O]^+^ 417 [M+H-C_8_H_6_O-CH_3_OH]^+^ 405 [M+H-C_9_H_6_O_3_]^+^ 167 [C_8_H_6_O_4_]^+^ 121 [C_7_H_6_O_2_]^+^	C_32_H_22_O_10_	Ginkgetin

8	11.47	567.1283	566.1212	567 [M+H]^+^ 435 [M+H-C_9_H_8_O]^+^ 417 [M+H-C_9_H_8_O-H_2_O]^+^ 391 [M+H-C_10_H_8_O_3_]^+^ 167 [C_8_H_6_O_4_]^+^ 135 [C_8_H_8_O_2_]^+^	C_32_H_22_O_10_	Putraflavone

9	14.92	581.1434	580.1369	581 [M+H]^+^ 449 [M+H-C_9_H_8_O]^+^ 417 [M+H-C_9_H_8_O-CH_3_OH]^+^ 405 [M+H-C_10_H_8_O_3_]^+^ 167 [C_8_H_6_O_4_]^+^ 135 [C_8_H_8_O_2_]^+^	C_33_H_24_O_10_	Heveaflavone
